# Sleep quality in women with nausea and vomiting of pregnancy: a cross-sectional study

**DOI:** 10.1186/s12884-021-03639-2

**Published:** 2021-02-19

**Authors:** Linda Laitinen, Miina Nurmi, Päivi Rautava, Mari Koivisto, Päivi Polo-Kantola

**Affiliations:** 1 Department of Obstetrics and Gynecology, Central Finland Central Hospital, Keskussairaalantie 19, 40620 Jyväskylä, Finland; 2 University of Turku, Turku, Finland; 3 Department of Public Health, University of Turku, Turku, Finland; 4 Turku Clinical Research Centre, Turku University Hospital, Turku, Finland; 5 Department of Biostatistics, University of Turku, Turku, Finland; 6 Department of Obstetrics and Gynecology, Turku University Hospital, Turku, Finland; 7 Sleep Research Center, University of Turku, Turku, Finland

**Keywords:** Nausea, Vomiting, Pregnancy, Sleep, Sleep disturbances

## Abstract

**Background:**

Nausea and vomiting of pregnancy (NVP) deteriorates many aspects of daily lives of women. However, little is known about associations between NVP and sleep quality.

**Methods:**

Women attending to routine mid-pregnancy visits in maternity health care clinics in Turku city area and surrounding municipalities, Finland, during 2011–2014, were invited to participate. A cohort of 1203 volunteers (mean age 30 years, mean gestational week 16.6, mean BMI 24.4 kg/m^2^, nulliparous 46%) was recruited. The severity of NVP in the worst 12-h period of current pregnancy was assessed with Pregnancy Unique Quantification of Emesis Questionnaire (PUQE) and categorized accordingly into no/mild/moderate and severe NVP. Sleep disturbances during the past 3 months were assessed with selected questions (difficulty falling asleep, night awakenings, too early morning awakenings and sleepiness during the day) from Basic Nordic Sleep Questionnaire (BNSQ). In addition, general sleep quality, as well as physical and mental quality of life (QoL) were rated with three visual analog scales (VAS). Associations between PUQE categories (severity of NVP) and sleep disturbances, general sleep quality, physical QoL and mental QoL were evaluated with multinomial regression analysis.

**Results:**

According to PUQE, NVP was most frequently moderate (*n* = 629, 52.3%), followed by mild (*n* = 361, 30.0%) and severe (*n* = 77, 6.4%). Only 11.3% had no NVP (*n* = 136). The most frequent sleep disturbance was night awakenings (69.9%, *n* = 837), followed by sleepiness during the day (35.7%, *n* = 427), too early morning awakenings (12.0%, *n* = 143) and difficulty falling asleep (7.1%, *n* = 81). In adjusted analysis (age, parity, body mass index, smoking, employment), more severe NVP was associated with night awakenings (AOR 3.9, 95% CI 1.79–8.47, *P* < 0.0001) and sleepiness during the day (AOR 4.7, 95% CI 2.20–9.94, *P* < 0.0001). In VAS, women with more severe NVP rated worse general sleep quality and worse physical and mental QoL. However, in multivariable analysis, the association between the severity of NVP and physical and mental QoL was stronger than that of sleep .

**Conclusions:**

More severe NVP is associated with sleep disturbances and in close relation to lower physical and mental QoL. Thus, in comprehensive care of women with NVP, also sleep quality should be evaluated.

## Background

Nausea and vomiting of pregnancy (NVP) manifests as various degrees of severity, at the worst as hyperemesis gravidarum (HG) [1, 2]. Even mild NVP has been shown to decrease women’s quality of life (QoL), activities of daily living and ability to perform family duties [3–6]. In addition, NVP typically causes absences from work [6] and women with HG often end up being repeatedly hospitalized [2].

Sleep quality is one cornerstone in QoL. Previous literature has described worsening of sleep quality and increase in sleep disturbances during pregnancy [7–9]. These changes in sleep begin already during the first trimester [7, 10, 11]. Sleep disturbances can be specified as sleep onset disturbances (difficulty falling asleep) and sleep maintenance disturbances (night awakenings, too early morning awakenings) [12–14], however, occurrence of these distinct sleep disturbances especially in early pregnancy are less studied [7, 9, 10, 13]. Despite this evident deterioration of sleep quality during pregnancy, little is known about the mechanisms and contributing factors. Mental health state has an important influence: depression and anxiety can worsen sleep quality but sleep disturbances may also induce mental symptoms or increase their severity [9, 15–17]. In addition, several physical discomforts often induce sleep disturbances during pregnancy [13, 18].

Previous studies evaluating associations between NVP and sleep quality are sparse [11, 13, 19, 20]. In a study by Mindell et al. [13], nausea was frequently reported as a symptom disturbing sleep, whereas Ertmann et al. [11], Ebrahimi et al. [19] and Heitmann et al. [20] found no association. Despite the term “morning sickness”, NVP symptoms are usually experienced throughout the day and also nighttime [21]. Therefore, we hypothesized that NVP may induce both sleep onset and sleep maintenance disturbances, and to assess them using only one question of general sleep quality in questionnaires is insufficient. Thus, our aim was to study the associations between the severity of NVP and specific sleep disturbances. To address this, we used a detailed sleep questionnaire and questionnaire for the severity of NVP.

## Methods

The study population of this cross-sectional study consisted of 2411 pregnant women who were recruited from 33 maternity health care clinics (MHCCs) in Turku city area and surrounding municipalities in Finland during their routine mid-pregnancy visits between October 2011 and November 2014. In Finland, more than 99% of pregnant women attend free of charge public MHCCs [22]. After receiving oral and written information about the study by the trained nurses, the volunteer women filled in the study questionnaire. Capability to understand Finnish language was required. Joint Ethics Committees of University of Turku and Turku University Central Hospital, Turku, Finland (58/180/2011) gave ethical approval. All participants gave a written informed consent. The study was conducted in accordance with the ethical standards as laid down in the 1964 Declaration of Helsinki and its later amendments.

The basic characteristics were collected from the Medical Birth Register of Finnish Institute for Health and Welfare, including parity (nulliparous/multiparous), pre-pregnancy body mass index (BMI, kg/m^2^) calculated by weight and height, smoking (no/yes), marital status (cohabited/single) and employment (working/not working). Age (years) was calculated by comparing the date of birth to the answering date. Nationality (Finnish/others) and gestational week (gwk) were gathered from the study questionnaire (Table 1).

**Table 1:** Basic characteristics. Total *n* = 1203

	n	Mean ± SD	Range
		or n (%)	
Age (years)	1194	30 ± 5	18–44
Gwk	1203	16.6 ± 2	7–20
Parity	1169		
Nulliparous		536 (45.9)	
Multiparous		633 (54.2)	
BMI (kg/m^2^)	1168	24.4 ± 4.8	16.7–57.8
Smoking	1166		
Non-smokers		1014 (87.0)	
Smokers		152 (13.0)	
Marital status	1157		
Cohabited		1115 (96.4)	
Single		42 (3.6)	
Employment	1029		
Working		847 (82.3)	
Not working		182 (17.7)	
Nationality	1190		
Finnish		1174 (98.7)	
Others		16 (1.3)	

*BMI* Body mass index, *Gwk* Gestational week

The severity of NVP was evaluated with Pregnancy-Unique Quantification of Emesis (PUQE) questionnaire [23] which has been validated also among Scandinavian population [24, 25]. With permission from the PUQE owners, the PUQE was translated into Finnish by professional translator and re-translated by another professional translator [26]. PUQE questionnaire rates NVP symptoms with three short questions: duration of nausea in hours and the quantity of both vomiting and retching episodes in a five-point scale, where higher points indicate more severe symptoms. PUQE total score ranges between 3 to 15 points and the questionnaire categorizes NVP into ‘no NVP’ (3 points), ‘mild’ (4–6 points), ‘moderate’ (7–12 points) and ‘severe NVP’ (≥13 points). In our study, the women were instructed to reply according to the worst 12 h of current pregnancy.

The primary outcomes included sleep quality measurements. General sleep quality during the worst 12-h period of NVP was measured with visual analog scale (VAS) of 0–100 where higher number indicated better sleep quality. The various sleep disturbances during the past 3 months were evaluated with Basic Nordic Sleep Questionnaire (BNSQ) [27]. In the BNSQ, the occurrence of sleep disturbances is rated with a five-point scale: 1 ‘never or less than once per month’; 2 ‘less than once per week’; 3 ‘on 1–2 nights per week’; 4 ‘on 3–5 nights per week’; 5 ‘every or almost every night’. In our study questionnaire, BNSQ questions concerning ‘difficulties falling asleep’, ‘night awakenings’, ‘too early morning awakenings’ and ‘sleepiness during the day’ were selected. The answers were dichotomized (yes: ≥3 times a week vs no: < 1–2 times a week) as previously established to represent clinically relevant sleep disturbances [28] and also used in studies of pregnant populations [9, 16].

As for secondary outcomes, estimations of physical and mental quality of life (QoL) during the worst 12-h period of NVP were measured with two different VAS of 0–100 where higher number indicated better QoL. In analysis, all VAS items were reversed and thus in our results higher VAS number indicated worse sleep quality and worse physical and mental QoL.

### Statistical analyses

Continuous variables were characterized using means, standard deviations (SD) and ranges and categorical variables using frequencies and percent. The severity of NVP was categorized by PUQE total score as no/mild/moderate and severe NVP. Frequencies and percentages of each PUQE category were calculated. According to questions from the BNSQ, frequencies and percentages of sleep disturbances (yes: ≥ 3 times a week vs no: < 1–2 times a week) in each PUQE category were calculated. Similarly, means, SD and ranges of general sleep quality and physical and mental QoL, measured by VAS scales, in each PUQE category were calculated. The assumptions for each statistical model were checked at first. Univariate associations between the severity of NVP (PUQE) and general sleep quality (VAS), sleep disturbances (BNSQ), physical QoL (VAS) and mental QoL (VAS) were calculated separately using multinomial logistic regression analysis. Further, all these analyses were adjusted for basic characteristics age, parity, BMI, smoking and employment. The characteristics for adjustment were selected because these variables have been shown to be associated with NVP in previous studies [1, 2] as well as with sleep disturbances during pregnancy [9, 16]. Thereafter, three different multivariable analyses (multinomial logistic regression analysis) were performed. First, model 1, between PUQE total score, sleep disturbances (BNSQ), basic characteristics (age, parity, BMI, smoking, and employment) and physical QoL. Secondly, model 2, between PUQE total score, sleep disturbances (BNSQ), basic characteristics and mental QoL. And finally, model 3, between PUQE total score, sleep disturbances (BNSQ), basic characteristics and both physical and mental QoL. General sleep quality (VAS) was not included in the multivariable models 1–3 because of interrelation with BNSQ variables. The results are presented with *p*-values, OR and 95% CI. Statistical analyses were carried out using a 9.4 version of SAS Institute Inc. (Cary, NC, USA) for Windows, and *p-*values of < 0.05 were considered as statistically significant.

## Results

### Clinical characteristics of the study population

**Fig. 1:**
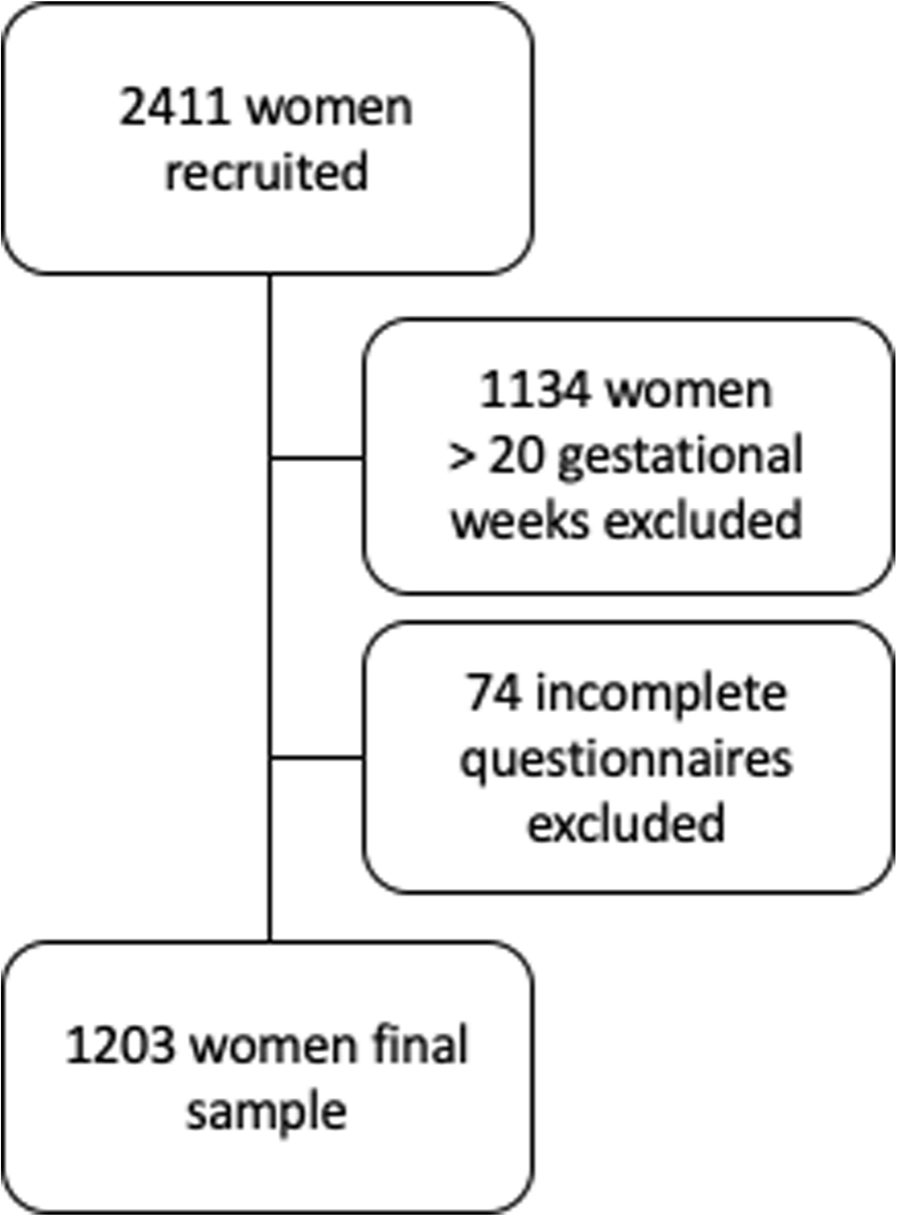
Flowchart of the study

To target the study period to the first and part of second trimester, women who responded after 20 gwk (*n* = 1134) were excluded. Furthermore, incomplete questionnaires (*n* = 74, missing data of PUQE/BNSQ/VAS/gwk/register data) were excluded, and thus 1203 women (mean aged 30 years [SD 5, range 18–44] years, mean gwk 16.6 [SD 2, range 7–20]) formed the final study cohort (Fig. 1). Basic characteristics of the women are presented in Table 1.

The women suffered most frequently from moderate NVP (52.3%). Of all women, mild NVP was reported by 30.0% and severe NVP by 6.4%. Only 11.3% reported no NVP. The most frequent sleep disturbance was night awakenings (69.9%,). In addition, 35.7% of the women reported sleepiness during the day and 12.0% too early morning awakenings. Only a smaller proportion of the women experienced difficulty falling asleep (7.1%) (Table 2).

**Table 2:** Sleep disturbancies, physical and mental QoL and associations with NVP. Total *n* = 1203

	All			No NVP		Mild NVP					Moderate NVP					Severe NVP				
				PUQE 3 points		PUQE 4–6 points					PUQE 7–12 points					PUQE ≥ 13 points				
	*n* = 1203			*n* = 136		*n* = 361					*n* = 629					*n* = 77				
	n (%)	*p*^*a*^	*p*^*b*^	n (%)	OR	n (%)	OR	95% CI	AOR^a^	95% CI	n (%)	OR	95% CI	AOR^a^	95% CI	n (%)	OR	95% CI	AOR^a^	95% CI
Difficulty falling asleep		0.005	0.026		1		1.05	0.37–2.98	1.00	0.30–3.18		2.66	1.04–6.75	2.46	0.86–7.07		3.01	0.95–9.57	3.00	0.80–11.29
Yes (≥ 3 times a week)	85 (7.1)			5 (0.4)		14 (1.1^c^)					58 (4.8)					8 (0.7)				
No (≤ 1–2 times a week)	1113 (92.9)			130 (10.9)		346 (28.9)					568 (47.4)					69 (5.8)				
Night awakenings		< 0.0001	< 0.0001		1		1.34	0.90–2.01	1.33	0.86–2.05		2.39	1.62–3.52	2.08	1.38–3.15		3.87	1.95–7.70	3.90	1.79–8.47
Yes (≥ 3 times a week)	837 (69.9)			75 (6.3)		227 (19.0)					471 (39.4)					64 (5.4)				
No (≤ 1–2 times a week)	360 (30.1)			59 (4.9)		133 (11.1)					155 (12.9^b^)					13 (1.0^b^)				
Too early morning awakenings		0.0008	0.013		1		2.02	0.82–4.95	1.81	0.72–4.53		3.82	1.64–8.91	3.16	1.33–7.49		3.97	1.42–11.04	3.05	0.99–9.37
Yes (≥ 3 times a week)	143 (12.0)			6 (0.5)		31 (2.6)					94 (7.9)					12 (1.00)				
No (≤ 1–2 times a week)	1053 (88.0)			129 (10.8)		330 (27.6)					529 (44.2)					65 (5.4)				
Sleepiness during the day		< 0.0001	< 0.0001		1		2.87	1.67–4.95	2.90	1.60–5.28		4.93	2.93–8.29	5.07	2.86–8.97		4.15	2.11–8.15	4.67	2.20–9.94
Yes (≥ 3 times a week)	427 (35.7)			18 (1.5)		110 (9.2)					269 (22.5)					30 (2.5)				
No (≤ 1–2 times a week)	768 (64.3)			117 (9.8)		249 (20.9^b^)					355 (29.7)					47 (3.9)				
	Mean ± SD			Mean ± SD		Mean ± SD					Mean ± SD					Mean ± SD				
General sleep quality VAS	53.5 ± 30.5	< 0.0001	< 0.0001	3.6 ± 9.1	1	40.7 ± 25.0	1.05	1.03–1.06	1.05	1.03–1.07	67.3 ± 21.7	1.07	1.05–1.09	1.07	1.06–1.09	81.6 ± 18.5	1.08	1.06–1.10	1.09	1.07–1.11
Physical QoL VAS	33.7 ± 28.4	< 0.0001	< 0.0001	3.6 ± 10.4	1	23.7 ± 21.7	1.17	1.13–1.20	1.20	1.16–1.25	43.0 ± 27.2	1.22	1.18–1.26	1.25	1.20–1.31	55.4 ± 30.2	1.28	1.23–1.32	1.32	1.26–1.39
Mental QoL VAS	31.4 ± 25.6	< 0.0001	< 0.0001	10.9 ± 17.5	1	25.0 ± 23.2	1.12	1.09–1.15	1.14	1.10–1.17	37.5 ± 25.0	1.15	1.12–1.19	1.17	1.13–1.21	45.5 ± 26.1	1.17	1.14–1.21	1.19	1.15–1.23

Odds ratios presented with 95% confidence interval

*AOR* Adjusted odds ratio, *BMI* Body mass index, *NVP* Nausea and vomiting of pregnancy, *PUQE* Pregnancy-Unique Quantification of Emesis questionnaire, Sleep disturbances were assessed with Basic Nordic Sleep Questionnaire, *QoL* Quality of life, *VAS* Visual analog scale

^a^Univariate analysis (multinomial logistic regression analysis)

^b^Adjusted for age, parity, BMI, smoking and employment (multinomial logistic regression analysis)

^c^Results rounded up with one decimal

### Associations between sleep disturbances and the severity of NVP

All in all, women with more severe NVP had more sleep disturbances. In univariate analysis, women with more severe NVP rated worse general sleep quality. As for distinct sleep disturbances, women with both moderate and severe NVP had higher odds for night awakenings and too early morning awakenings. Further, moderate NVP was associated with difficulties falling asleep. In addition, NVP in all severity levels was associated with sleepiness during the day (Table 2).

After adjusting the results for age, parity, BMI, smoking and employment, both moderate and severe NVP were associated with night awakenings, and moderate NVP with too early morning awakenings. Furthermore, NVP in all severity levels was associated with sleepiness during the day (Table 2.)

### Associations between QoL and the severity of NVP

**Fig. 2:**
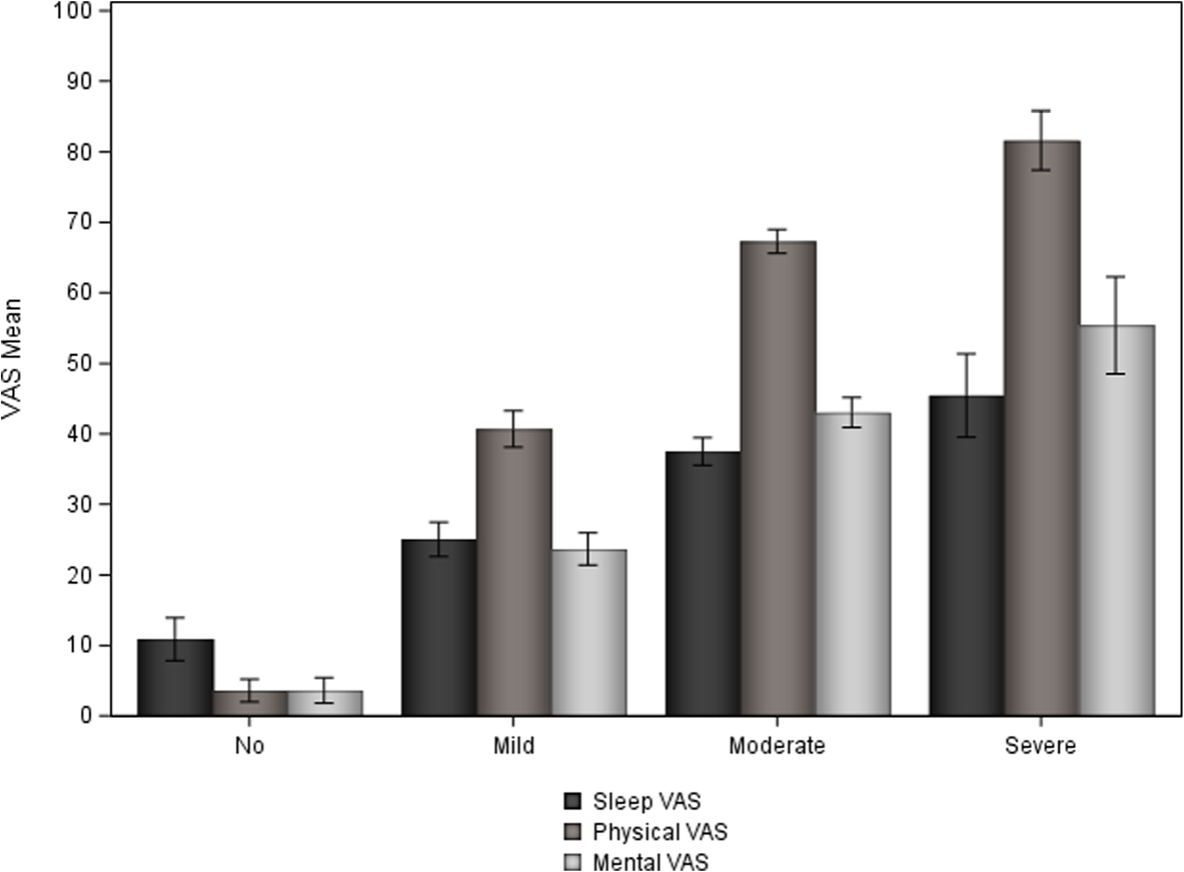
General sleep quality, physical and mental QoL in women with NVP. Presented as means and confidence intervals. Higher score in scales indicates worse quality: the worse the NVP the worse general sleep quality and physical and mental QoL. NVP = Nausea and vomiting of pregnancy. QoL = Quality of life. VAS = Visual analog scale

The women with more severe NVP reported worse physical and worse mental QoL accordingly. These findings remained the same after adjusting for age, parity, BMI, smoking and employment. (Fig. 2 and Table 2).

### Associations between sleep disturbances, QoL and the severity of NVP

In multivariable model 1 including PUQE total score, sleep disturbances (BNSQ), physical QoL and basic characteristics (age, parity, BMI, smoking and employment), worse physical QoL was associated with all severity levels of NVP, whereas the sleep disturbances lost their associations with NVP. In multivariable model 2 including PUQE total score, sleep disturbances (BNSQ), mental QoL and basic characteristics, both moderate and severe NVP were associated with nighttime awakenings and moderate NVP with sleepiness during the day. Furthermore, worse mental QoL was associated with all severity levels of NVP. (Table 3). In multivariable model 3, including PUQE total score, sleep disturbances (BNSQ), physical and mental QoL and basic characteristics, both worse physical QoL (*p* < 0.0001; mild NVP AOR 1.19, 95% CI 1.14–1.25, moderate NVP AOR 1.23, 95% CI 1.18–1.29, severe NVP AOR 1.30, 95% CI 1.24–1.37) and worse mental QoL (*p* = 0.007; mild NVP AOR 1.03, 95% CI 1.00–1.08, moderate NVP AOR 1.04, 95% CI 1.00–1.08, severe NVP AOR 1.05, 95% CI 1.01–1.09) were associated with more severe NVP, whereas sleep disturbances lost their associations with NVP.

**Table 3:** Multivariable associations between sleep disturbances, physical and mental QoL and the severity of NVP. Total *n* = 1203

			No NVP	Mild NVP				Moderate NVP				Severe NVP			
			PUQE 3 points	PUQE 4–6 points				PUQE 7–12 points				PUQE ≥ 13 points			
			*n* = 136	*n* = 361				*n* = 629				*n* = 77			
	*p*^*a*^	*p*^*b*^	AOR	AOR^a^	95% CI	AOR^b^	95% CI	AOR^a^	95% CI	AOR^b^	95% CI	AOR^a^	95% CI	AOR^b^	95% CI
Difficulty falling asleep	0.367	0.466	1	0.26	0.03–1.92	0.33	0.08–1.39	0.46	0.06–3.54	0.49	0.12–2.00	0.43	0.04–4.21	0.48	0.09–2.59
Night awakenings	0.421	0.011	1	0.77	0.39–1.52	1.30	0.79–2.14	0.88	0.42–1.84	1.84	1.10–3.09	1.42	0.51–3.96	3.52	1.49–8.35
Too early morning awakenings	0.730	0.842	1	0.50	0.10–2.51	0.62	0.21–1.89	0.50	0.10–2.64	0.63	0.21–1.92	0.36	0.06–2.29	0.55	0.14–2.17
Sleepiness during the day	0.321	0.024	1	1.67	0.60–4.63	2.37	1.16–4.83	1.72	0.60–4.95	2.88	1.41–5.90	1.06	0.32–3.52	2.04	0.82–5.10
Physical QoL VAS	< 0.0001	NA	1	1.21	1.16–1.26	NA	NA	1.26	1.20–1.31	NA	NA	1.33	1.27–1.40	NA	NA
Mental QoL VAS	NA	< 0.0001	1	NA	NA	1.13	1.10–1.17	NA	NA	1.16	1.12–1.20	NA	NA	1.19	1.15–1.23

Odds ratios presented with 95% confidence interval

*AOR* Adjusted odds ratio, *BMI* Body mass index, *NA* Not applicable, *NVP* Nausea and vomiting of pregnancy, *PUQE* Pregnancy-Unique Quantification of Emesis questionnaire, Sleep disturbances were assessed with Basic Nordic Sleep Questionnaire, *QoL* Quality of life, *VAS* Visual analog scale

^a^Model 1: PUQE score, sleep disturbances, physical QoL, basic characteristics (age, parity, BMI, smoking, employment) (multinomial logistic regression analysis)

^b^Model 2: PUQE score, sleep disturbances, mental QoL, basic characteristics (age, parity, BMI, smoking, employment) (multinomial logistic regression analysis)

## Discussion

Our study confirmed the findings of the few previous reports of an association between worse general sleep quality and NVP. Using a detailed sleep questionnaire, we further showed that disturbances in sleep maintenance were associated with NVP, mostly in a dose response manner; the worse the NVP the higher the odds for sleep disturbances. Furthermore, women with even mild NVP suffered from sleepiness, presumably indicating that sleepiness does not stem only from sleep disturbances but also from pregnancy per se.

Previously, only few studies have evaluated associations between NVP and sleep quality. Ebrahimi et al. [19] studied sleep quality by calculating the total reported hours of sleep as part of the validation study of PUQE–24 questionnaire (*n* = 311 women) and found no associations with NVP. In a population-based Norwegian study [20] with 712 presently pregnant or recently delivered women, occurrence of sleep problems was 53–64% regardless of the severity of NVP. In their web-based questionnaire, women could simply tick a box of existence of sleep problems. Contrary to these studies, using one VAS question as a measure of general sleep quality, we found an association between the severity of NVP and worse sleep quality. The different outcomes in these studies could at least partly be explained by the different formulations of the questions concerning general sleep quality. We used a VAS question, which is a generally established tool to assess various sensations and symptoms in medicine [29].

Sleep disturbances form a combination of symptoms, for the estimation of which structured and validated questionnaires bring accuracy and diversity. In a cohort study by Mindell et al. [13], designed to estimate sleep patterns and sleep disturbances with Pittsburgh Sleep Quality Index in all months of pregnancy in 2427 women, nausea was frequently reported as a symptom disturbing sleep in the first 3 months of pregnancy. On the contrary, Ertmann et al. [11] studied 1338 women for sleep complaints in early pregnancy with questions from Nottingham Health Profile (long time to fall asleep, waking up too early and lying awake most of the night) and associated pregnancy-related physical symptoms. In their study, waking up too early was the most prevalent sleep complaint (47%) but nausea or vomiting were not associated with any sleep complaints in the multivariable analysis which included physical and mental health status. However, in the adjusted analysis (age, sociodemographic characteristics and reproductive background), nausea was associated with moderate and severe sleep complaints. We utilized validated BNSQ [27], which has previously been used also in pregnant populations [9, 16]. In our study, more severe NVP was associated especially with sleep maintenance disturbances: both nighttime awakenings and too early morning awakenings were increased.

The importance of sleep disturbances is pronounced by their daytime consequences, fatigue, tiredness and sleepiness. In sleep medicine, there is actually a clear difference in definitions of these terms, nevertheless, in the literature they are often mixed. Fatigue signifies exhaustion related to insomnia and feeling of sub alertness, tiredness is related to decreased freshness and vigor, whereas sleepiness indicates drowsiness and is typically connected to sleep loss [30]. According to some previous studies, fatigue is shown to correlate with nausea in early pregnancy [31–33]. As for tiredness, in early pregnancy it may be promoted by several hormonal factors [34]. Of daytime consequences, we evaluated sleepiness and found that women with more severe NVP suffered more daytime sleepiness. This could be expected, since more severe NVP was also associated with night awakenings and too early morning awakenings, which typically cause sleep loss [35] and thus may be reflected as sleepiness [36].

Previous studies have confirmed that NVP decreases QoL [33, 37]. In our study, too, women with more severe NVP rated worse physical and mental QoL. Sleep quality is important in connection with both physical and mental QoL [38]. Furthermore, the associations are bilateral. Physical symptoms and diseases decrease sleep quality [18] and sleep disturbances induce physical symptoms as well [35]. The same holds true also between mental symptoms and diseases and sleep quality [15–17, 39]. In our study, this interrelation between mental QoL and specific sleep disturbances was reflected in multivariable analysis of women with more severe NVP.

Our study has some limitations. First, we enquired women to evaluate NVP symptoms during the worst 12-h period of current pregnancy, but 24-h scale would have covered also nighttime more accurately. In addition, we did not record the exact gwk of the occurrence of the worst 12-h period of NVP. Since the women were recruited between 7 to 20 gwk it is possible that some women in early gwk had not yet reached their worst period of NVP and, on the other hand, some women had already recovered weeks ago. In most women NVP resolves at least after 20 gwk [21] and as the mean gwk in our study was 16.6, it presumably covered the main occurrence of NVP in most of the women. Secondly, NVP treatment includes medications, specifically antihistamines, which can induce tiredness. Unfortunately, we did not have the information of the medication used by our study participants. However, in Finland, the first line medication in NVP is metoclopramide and antihistamines are not used in this indication. Thereafter the number of women with sedative treatment could be estimated to be very low. Third, because the data of NVP symptoms and sleep quality were collected from questionnaires instead of health records, we were not able to evaluate the associations between NVP, sleep and health status. In addition, the pre-pregnancy sleep quality and mental health state were not inquired. Therefore, we were not able to exclude that the association between NVP and sleep disturbances were dependent on pre-pregnancy sleep disturbances or mental health problems. Fourth, only voluntary women were recruited, the women were not consecutive clients. Thus, it is possible that only women with worse NVP or more sleep disturbances may have been overrepresented. However, the percentage of women with no NVP was higher than those with severe NVP. Moreover, the frequency of sleep disturbances fell into the similar range as previously reported in pregnant populations [9, 16, 39]. Fifth, the questionnaires of 74 women were excluded from the study because of incomplete or missing data, hence we were unable to carry out the drop-out analysis. Further, we lack the full record of reasons for non-participation and the exact number of women who refused to participate.

The enrollment from public free of charge MHCCs, in which practically all pregnant women attend in Finland, could be considered as a merit. Thus, the women in our study represented general Finnish pregnant population which is quite homogenous. In addition, this kind of study protocol enabled us to recruit women with different severity of NVP and also asymptomatic women. To minimize recall bias of the severity of NVP, only women responding in the first and early second trimester were included in the analysis. Further strengths were a high number of participants and the use of validated questionnaires, PUQE and BNSQ [23, 27]. The BNSQ inquires sleep disturbances during the past 3 months and as mean gestational age of the women was 16.6 gwk, this period of 3 months covered the first trimester in most of the women. In addition, as QoL and sleep quality are closely related, the self-reported physical and mental QoL assessments were included in the analysis. Moreover, the results were adjusted for basic characteristics of age, parity, BMI, smoking and employment because in previous studies these confounders have been linked with NVP [1, 2] as well as with sleep quality during pregnancy [9, 16].

## Conclusions

According to our study, using a detailed sleep questionnaire, we found that more severe NVP was associated with sleep maintenance problems. Further, women suffering from more severe NVP had not only worse physical and mental QoL but also worse general sleep quality. In clinical care, the importance of sleep quality as part of women’s QoL should be acknowledged when treating women with NVP.

## Data Availability

The datasets generated and analyzed during the current study are available from the corresponding author on reasonable request.

## Abbreviations

AOR: Adjusted odds ratio
BNSQ: Basic Nordic Sleep Questionnaire
CI: Confidence interval
GWK: Gestational week
MHCC: Maternity health care clinic
NVP: Nausea and vomiting of pregnancy
OR: Odds ratio
PUQE: Pregnancy Unique Quantification of Emesis Questionnaire
QoL: Quality of life
VAS: Visual analog scale
